# Swin Unet3D: a three-dimensional medical image segmentation network combining vision transformer and convolution

**DOI:** 10.1186/s12911-023-02129-z

**Published:** 2023-02-14

**Authors:** Yimin Cai, Yuqing Long, Zhenggong Han, Mingkun Liu, Yuchen Zheng, Wei Yang, Liming Chen

**Affiliations:** 1 School of Medical, Guizhou University, Guiyang, China; 2 School of Stomatolog, ZunYi Medical University, Zunyi, China; 3 Key Laboratory of Advanced Manufacturing Technology of Ministry of Education, Guizhou University, Guiyang, China; 4 Guiyang Dental Hospital (Dental Hospital of Guizhou University), Guizhou University, Guiyang, China

**Keywords:** Deep learning, Medical image segmentation, 3D Swin Transformer, Brain tumor

## Abstract

**Background:**

Semantic segmentation of brain tumors plays a critical role in clinical treatment, especially for three-dimensional (3D) magnetic resonance imaging, which is often used in clinical practice. Automatic segmentation of the 3D structure of brain tumors can quickly help physicians understand the properties of tumors, such as the shape and size, thus improving the efficiency of preoperative planning and the odds of successful surgery. In past decades, 3D convolutional neural networks (CNNs) have dominated automatic segmentation methods for 3D medical images, and these network structures have achieved good results. However, to reduce the number of neural network parameters, practitioners ensure that the size of convolutional kernels in 3D convolutional operations generally does not exceed $7 \times 7 \times 7$, which also leads to CNNs showing limitations in learning long-distance dependent information. Vision Transformer (ViT) is very good at learning long-distance dependent information in images, but it suffers from the problems of many parameters. What’s worse, the ViT cannot learn local dependency information in the previous layers under the condition of insufficient data. However, in the image segmentation task, being able to learn this local dependency information in the previous layers makes a big impact on the performance of the model.

**Methods:**

This paper proposes the Swin Unet3D model, which represents voxel segmentation on medical images as a sequence-to-sequence prediction. The feature extraction sub-module in the model is designed as a parallel structure of Convolution and ViT so that all layers of the model are able to adequately learn both global and local dependency information in the image.

**Results:**

On the validation dataset of Brats2021, our proposed model achieves dice coefficients of 0.840, 0.874, and 0.911 on the ET channel, TC channel, and WT channel, respectively. On the validation dataset of Brats2018, our model achieves dice coefficients of 0.716, 0.761, and 0.874 on the corresponding channels, respectively.

**Conclusion:**

We propose a new segmentation model that combines the advantages of Vision Transformer and Convolution and achieves a better balance between the number of model parameters and segmentation accuracy. The code can be found at https://github.com/1152545264/SwinUnet3D.

## Background

A brain tumor is an abnormal growth of cells in human brain tissue. When the tumor grows gradually, it presses on the affected nerves, causing a series of adverse symptoms and even threatening life. Brain tumors are medically classified into benign and malignant tumors, the latter of which are further classified into primary and metastatic brain tumors [1]. Primary brain tumors are initially lesion cells inside human brain tissue, while metastatic brain tumors are caused by cancer cells from other tissues that metastasize to the brain. Metastatic brain tumors are more common than primary brain tumors, and about half of metastatic tumors come from lung cancer [1]. Under the current scientific and technological conditions, it is no longer difficult to obtain three-dimensional (3D) imaging of brain tissue, whether by computed tomography (CT) or magnetic resonance imaging (MRI), but the problem mainly lies in how to reliably and quickly identify the tumor lesion area in the imaging and obtain the 3D spatial structure of the tumor so as to improve the efficiency of preoperative planning and the odds of successful surgery. One solution is to rely on doctors specialized in imaging to identify and outline the lesion areas in the images, but this method is too inefficient and requires a lot of time and labor. Because of the high computing speed of computers, it is a good choice to automate medical image segmentation with the help of image segmentation techniques in computer vision.

Image segmentation is the grouping of each pixel in an image into a certain category. This is the basis for understanding the concept of a scene [2]. Image segmentation plays a pivotal role in medical image analysis. Image segmentation can automatically outline the structure of diseased or other target tissues, providing information for subsequent diagnosis and treatment by physicians. Early medical image segmentation algorithms mainly include threshold-based segmentation algorithms [3], region-based segmentation algorithms [4], wavelet analysis and transform-based segmentation algorithms [5], Markov random field-based-based segmentation algorithms [6], and genetic algorithm-based segmentation algorithms [7]. With the improvement of hardware capability and the development of deep convolutional neural networks (CNNs), CNN has achieved dominance in the field of computer vision. The seminal U-net [8] network was proposed and achieved impressive results in the field of two-dimensional (2D) medical image segmentation. The classical Unet [8, 9] network consists of an encoder and a decoder; the encoder extracts image features using a series of convolutional and downsampling layers, while the decoder uses a series of transposed convolutional layers [10, 11] to upsample image features to the original image resolution for semantic prediction of pixels, in addition to incorporating the image features extracted by the encoder during the upsampling process to reduce the information loss during downsampling. Given the simple structure and superior performance of U-Net, scholars have subsequently proposed various variants of the U-Net model [9, 12–17].

Given the powerful fitting capability of such CNN-based neural networks, they have achieved superior results in various medical image segmentation tasks so far. However, in order to improve the computational speed of the model and reduce the number of parameters in the model, we must ensure that the maximum size of the convolutional kernels in most models does not exceed $3\times 3\times 3$, such as 3D-Unet [9], DenseVoxNet [18], and 3D $U^2-Net$ [19]. There are also some networks that use $5\times 5\times 5$ convolutional kernels, such as V-Net [14], and some networks that use $7\times 7\times 7$ convolutional kernels, such as ResNetMed3D [20]. The receptive field of CNN-based methods is highly localized due to the limitation of the fixed receptive field of the convolutional kernel. Although the perceptual field of these methods can gradually increase in higher layers, they still cannot fully learn long-range dependent information in lower layers [21, 22]. Nevertheless, such long-distance dependencies are crucial for accurate segmentation of tissue structures in medical images [19]. Some studies have attempted to overcome the CNN’s inability to acquire remote dependencies through techniques such as dilation convolution [23], the spatial attention mechanism, the channel attention mechanism [24], and the image pyramid [25], but these techniques still have some limitations. Inspired by the great success of Transformer [26] in the field of natural language processing, some scholars have introduced Transformer into the field of computer vision and have proposed Vision Transformer (ViT) [27] and Swin Transformer [28]. Dividing 2D images into image blocks and combining them with positional encoding, ViT achieves comparable performance with the CNN on large datasets. Swin Transformer proposes a window attention mechanism and a cyclic moving window attention mechanism to solve the problem of high time complexity in the computation of ViT. Based on the robust performance achieved by ViT on image classification tasks, some scholars have introduced it to image segmentation tasks. Swin-Unet [29] is a pure Transformer network structure, where the encoder and decoders are composed of Transformers. However, Swin-Unet is a model for 2D medical image segmentation, which is not applicable to voxel segmentation of 3D medical images unless a lot of additional work has been performed or some complex adaptation code has been written. TransUnet [21] and TransBTS [30] are a kind of hybrid model in combining CNN and Transformer, using successive convolutional layers and Transformer in the encoder for feature extraction and transposed convolution for upsampling operations in the decoder to recover spatial resolution for semantic segmentation. In the UnetR [31] and SwinBTS [32] structure, the authors used the Transformer to initially extract the image features while using the CNN as the backbone network in both its encoders and decoders.

Among the existing models, segmentation models implemented entirely based on CNNs have significant limitations in modeling long-distance dependent information in images, while segmentation models based entirely on ViT are unable to learn low-level detail information in images well [21, 22]. There are also some models that combine the advantages of ViT and CNN to model both long-distance dependent information and short-distance dependent information in images, but these models have a large number of model parameters and high computational time complexity due to the influence of ViT [28].

**Fig. 1:**
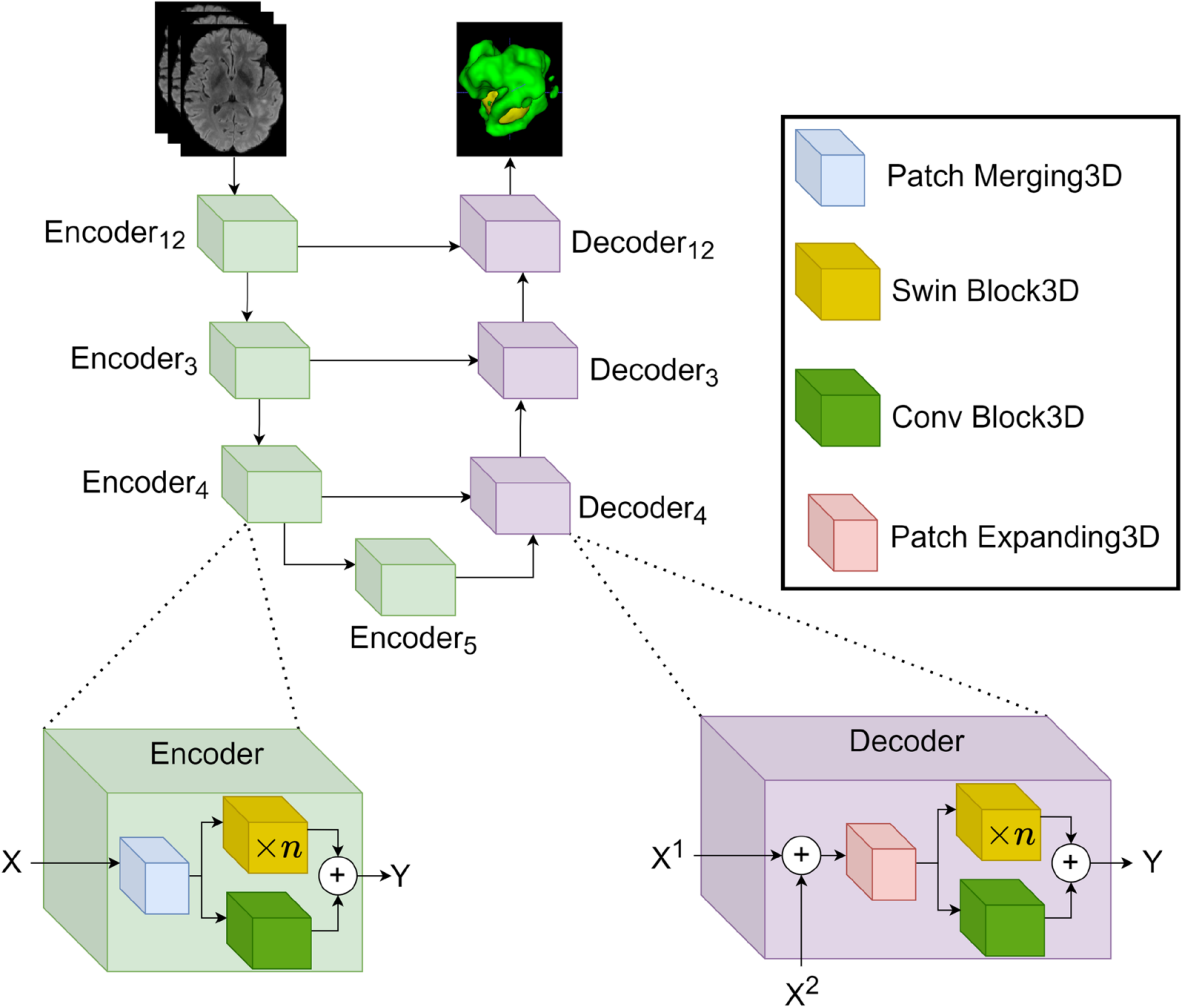
The architecture of the Swin Unet3D

Based on the above state of affairs and inspired by the work of Swin Transformer [28] and Swin Unet [29], we propose a new segmentation model, Swin Unet3D, for voxel segmentation of 3D medical images. Our model is proposed after consideration of the advantages and disadvantages of Swin Transformer and the CNN, and without degrading the model modeling capability, our model only adds additional linear time complexity compared to CNN-based segmentation models. Our model consists of an encoder, a decoder, and a jump connection, as shown in Fig. 1. A 3D medical image with a resolution of $H\times W\times D$ is divided into non-overlapping voxel patches of size $4\times 4\times 4$. Each voxel patch is then flattened by a fully connected layer and encoded as a 96-dimensional vector. Each vector is considered as a token, and these obtained $\frac{H}{4} \times \frac{W}{4} \times \frac{D}{4}$ tokens are then fed into a transformer-based encoder for image feature extraction. The image features extracted by the four encoders are sent to the decoder for upsampling, which recovers the spatial resolution of the image and gradually fuses them with the features extracted by the encoders to complete the semantic segmentation of the image using a jump connection. The experiments that have been conducted on the Brats2021 challenge dataset [33–35] and the Brats2018 challenge dataset [33–35] show that our model achieves a good balance in terms of segmentation accuracy and the number of model parameters.

Our contributions can be summarized as follows: (1) based on the idea of Swin Transformer, we first implemented Swin Transformer Block3D, a module that can extract features in 3D medical images like Convolution3D; (2) we proposed ViT and CNN parallel structures. The Swin Transformer-based Swin Block3D module is responsible for learning long-distance dependency information in images, while the CNN-based Conv Block3D is responsible for learning short-distance dependency information in images, and at the end of each decoder, the model performs feature fusion of the image features extracted by these two modules; (3) the results of ablation experiments show that in an intensive prediction task like image segmentation, the ViT structure and the Convolution structure combined can compensate for each other’s shortcomings.

## Methods

### Implementation details of Swin Unet3D

#### Architecture overview

Figure 1 presents an overview of Swin Unet3D, a model consisting of an encoder, a jump connection, and a decoder. The Patch Merging3D module is mainly used for image downsampling, while the Swin Transformer Block3D module and the Conv Block3D module are designed to extract image features. Specifically, Swin Block3D is employed to learn the long-range dependency information in the image, and Conv Block3D is adopted to learn local dependency information in the image. The patch Expanding3D module is used for upsampling to recover the spatial resolution of the image. A Patch Merging and several Swin Block3D are stacked to form a Down Stage, while a Patch Expanding3D and several Swin Block3D are stacked to form an Up Stage.

The input 3D image is first chopped into multiple $4 \times 4 \times 4$ voxel blocks, and then each voxel block is flattened into a one-dimensional vector of length 64. Finally, these one-dimensional vectors are linearly transformed and their length is changed to N. After completing the above steps, the input image is encoded as $\frac{H}{4} \times \frac{W}{4} \times \frac{D}{4}$ tokens, each tokens is a one-dimensional vector of length N. Referring to the original Swin Transformer [28], N can be set to 96. These tokens are further fed into the Conv Blocks3D module and Swin Block3D module for extracting the features of the image. The Patch Expanding3D module is used in the decoder to recover the spatial resolution of the feature vector, and the Swin Block3D and Conv Block3D modules are used to continue the feature extraction. After stacking multiple decoders, the spatial resolution of the feature map can be restored to the input spatial resolution, and the pixel-level segmentation prediction of the input image can be obtained by applying a linear change to the last layer of the feature map.

Each encoder or decoder contains a multi-header attention mechanism layer. According to the design of Swin Unet [29], the number of multi-headed attention mechanisms used by $Encoder_{12}, Encoder_3, Encoder_4$, and $Encoder_5$ are 3,6,9,12 respectively, and the number of multi-headed attention mechanisms used by $Decoder_4, Decoder_3, and Decoder_{12}$ is 9,6,3 respectively. The number n of Swin Blocks3D contained in each Encoder in Fig. 1 is 2,2,4,2 from $Encoder_{12}$ to $Encoder_5$, and 4,4,2 from $Decoder_4$ to $Decoder_{12}$, respectively.

**Fig. 2:**
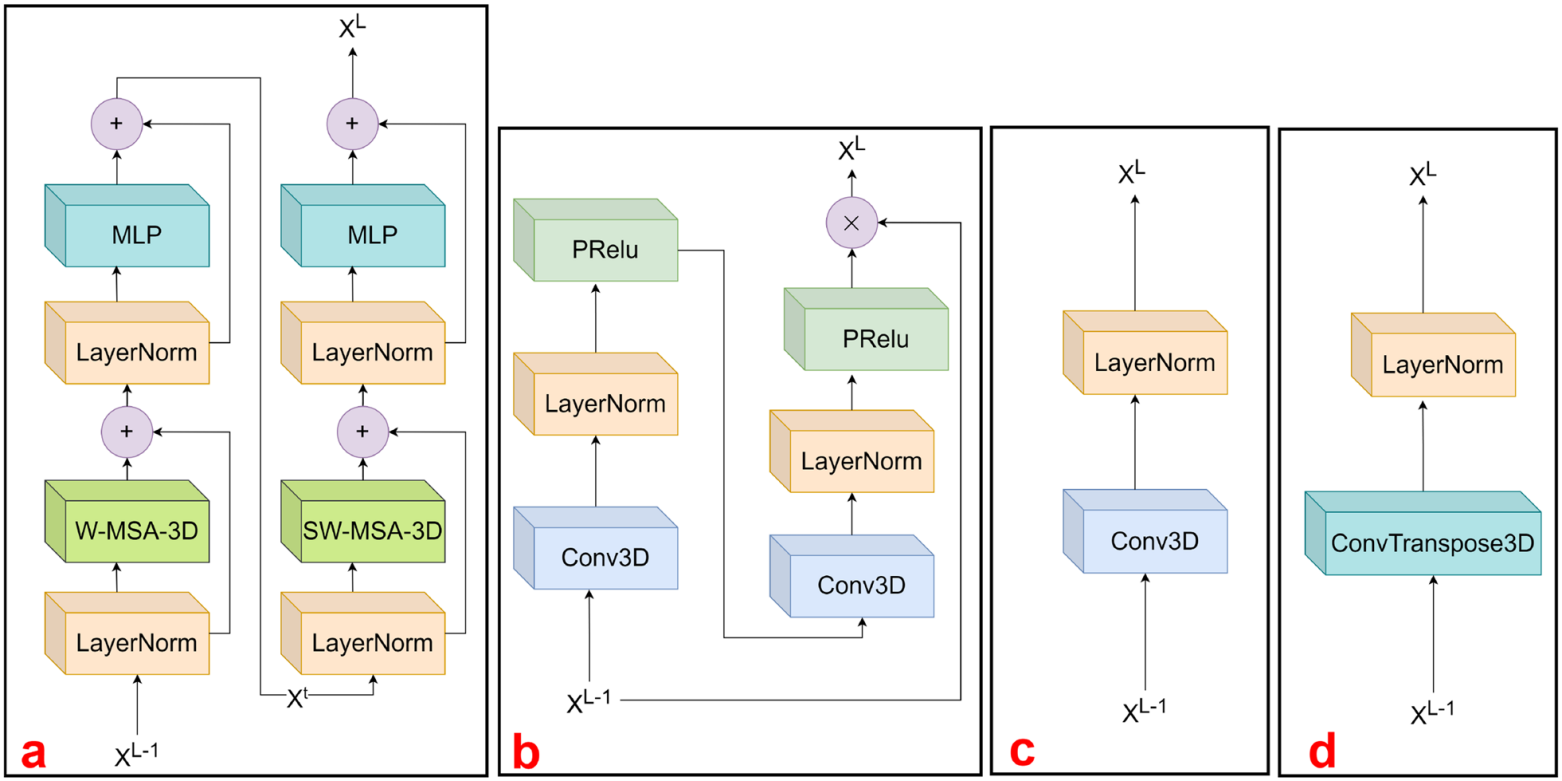
Overview of the structure of some sub-modules: **a** swin Block3D, **b** Conv Block3D, **c** Patch Merging3D, **d** Patch Expand3D

#### Multi-head window self-attention3D

The window multi-head self-attention3D (SW-MSA3D) module divides the input tokens into multiple sub-windows. The window size is specified according to the spatial resolution of the input image, which is generally equal to the spatial resolution of the input image divided by 32. The reason for using this design is that the images are downsampled four times during the encoding process, and the downsampling factors are 4, 2, 2, and 2. To avoid misalignment of feature map dimensions during the fusion using feature map information, it is necessary to set the spatial resolution of the input image to a multiple of 32. Also, to be able to divide the input image into multiple windows of the exact same size, we must be able to integer divide the input in each dimension by the size of the window in the corresponding dimension (Fig. 2).

**Fig. 3:**
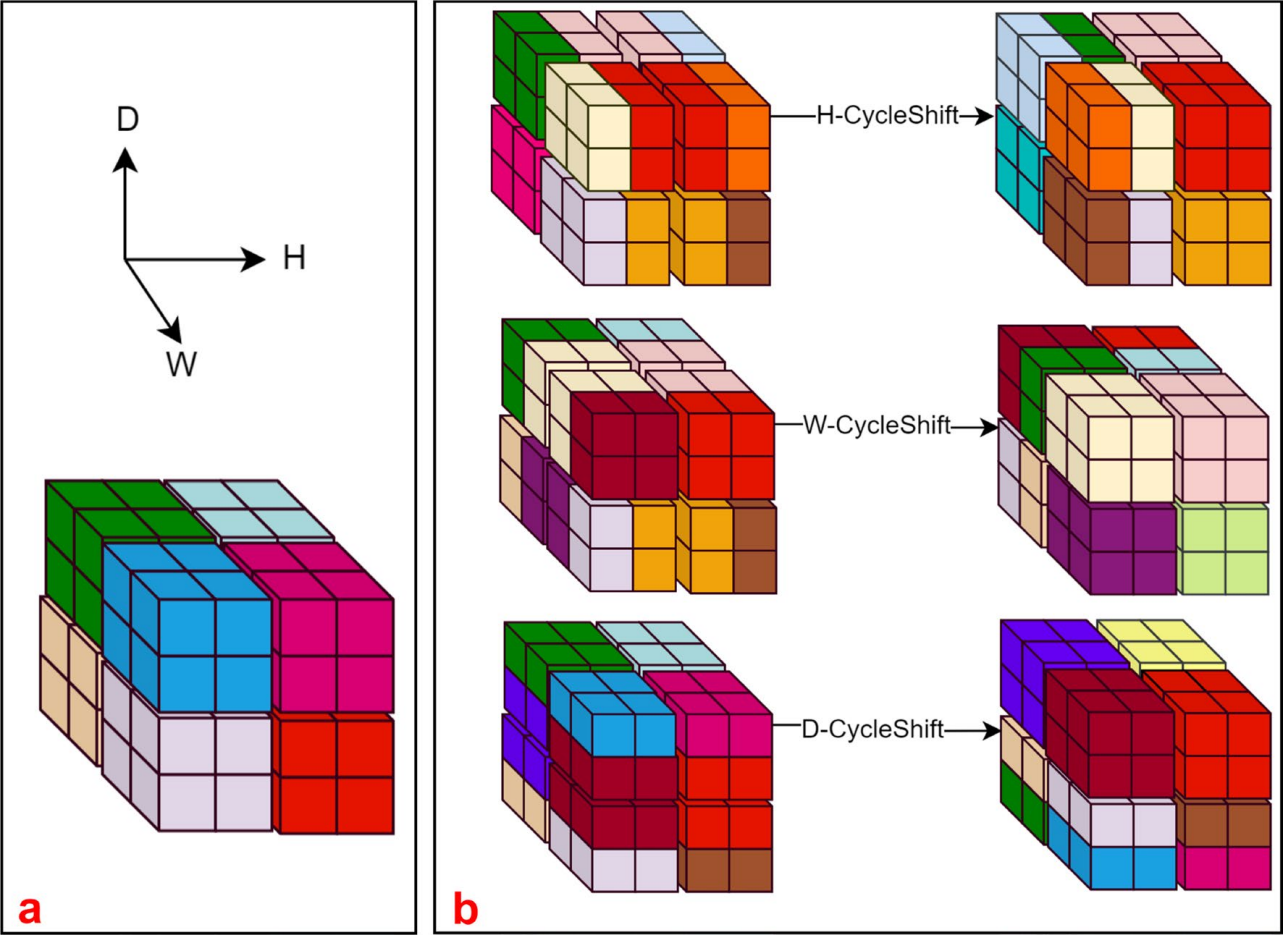
The schematic diagram of **a** W-MSA-3D and **b** SW-MSA-3D with a window size of 2. The tokens of the same color in **a** belong to the same window, and we only calculate the self-attention within each window. To obtain the dependency information interaction between adjacent windows, we divide some tokens within neighboring windows into the same window after cyclic shifting, and only tokens satisfying these conditions are allowed to calculate the window self-attention between them. Other tokens that do not satisfy the condition are shielded from attention between them by a masking mechanism even if they belong to the same window after a circular shift, as shown in **b**

Like the two-dimensional W-MSA, W-MSA-3D computes a multi-head self-attention mechanism for each window. It computes the similarity between the tokens in each window, and we adapted the idea of SW-MSA implementation in Video Swin Transformer [36] in the process of our implementation. However, as W-MSA-3D only calculates the similarity between tokens within the same window, it lacks the information interaction between windows. To solve this problem, the SW-MSA-3D mechanism is introduced. The input is cyclically shifted by *s* units in each dimension, where the value of *s* must be smaller than the window size in the corresponding dimension, and the default value of *s* is half of the window size with reference to the setting of the original paper [28]. However, the circular shifted-window mechanism leads to two more problems: (1) an increase in the number of windows; (2) inconsistent window sizes. Some tokens originally located in non-adjacent windows are in the same window after cyclic shifting, and the similarity generated between these tokens should be filtered out when calculating the self-attention inside the window, so we introduce the window-masking mechanism. Figure 3a shows the normal method of calculating the window self-attention, which only calculates the similarity between tokens of the same color, as they are in the same window. Figure 3b depicts the cyclic shifted-window mechanism. Tokens that are originally in adjacent windows (patches with the same color in Fig. 3b) are in the same window after cyclic shifting, and the similarity between them can be calculated. The similarity between tokens that are not in adjacent windows (patches with different colors in Fig. 3b) should be filtered out even if they are in the same window after the cyclic shifting. The inclusion of the cyclic shifted-window mechanism can introduce information interaction between neighboring windows with the cost of only increasing the linear computational complexity, thus allowing the neural network to learn long-distance dependency information. The process of calculating the attention between tokens in each window can be described by the following formula:

1 $$\begin{aligned} Q&= W^Q \times X \end{aligned}$$

2 $$\begin{aligned} K&= W^K \times X \end{aligned}$$

3 $$\begin{aligned} V&= W^V \times X \end{aligned}$$

4 $$\begin{aligned} attn&= Softmax\,\left(\frac{Q\times K^T}{\sqrt{d_k}}\right) \times V \end{aligned}$$

Each of$W^Q$,$W^K$,$W^V$ is a parameter-learnable square array with the same dimensionality, $d_k$ is the dimensionality of K, X is the matrix of tokens in the same window, and attn is the similarity between tokens.

#### Conv Block3D

The Conv Block3D module, shown in Fig. 2b, is stacked twice in the order of $1 \times 1\times 1$ convolutional layers, LayerNorm [37] layers, and PRelu [38] layers, and it is mainly responsible for learning the local dependencies of the images. The computational process of this module can be described as follows:

5 $$\begin{aligned} X^t&= PReLu_1(LN_1(Conv3D_1(X))) \end{aligned}$$

6 $$\begin{aligned} X^t&= PReLu_2(LN_2(Conv3D_2(X^t))) \end{aligned}$$

7 $$\begin{aligned} Y&= X^t \times X \end{aligned}$$

where X denotes the input of Conv Block3D, Y indicates the output of Conv Block3D, and $X^t$ denotes the intermediate temporary variable. In order to avoid the extra-large computational effort caused by this module, we use the depth-wise separable convolution [39] instead of the normal convolution. This sub-module was designed to ensure that the model can better fit the detailed information in the image and draws on the implementation in VAN [40] so that multiplication rather than addition is used in performing the feature convergence of $X^t$ and X.

#### Swin Block3D

The composition of Swin Block3D is shown in Fig. 2a, and its design idea is derived from the Block module in Swin Transformer [28]. It consists of two basic units: The first unit consists of a LayerNorm (LN) layer, window multi-Head self-attention3D (W-MSA-3D) module, a LayerNorm layer, and an MLP module in order of successive composition; the second unit uses the shifted-window multi-head self-attention3D (SW-MSA-3D) module, which replaces the W-MSA-3D module in the first cell, and the rest of the structure is the same as that of the first unit. The whole calculation process of Swin Block3D can be described by the following mathematical equation:

8 $$\begin{aligned} X^{t_1}&= LN_1(X^{L-1}) + W\text{- }SA\text{- }3D(LN_1(X^{L-1})) \end{aligned}$$

9 $$\begin{aligned} X^t&= LN_2(X^{t_1}) + MLP(LN_2(X^{t_1})) \end{aligned}$$

10 $$\begin{aligned} X^{t_2}&= LN_3(X^{t}) + SW\text{- }MSA\text{- }3D(LN_3(X^{t})) \end{aligned}$$

11 $$\begin{aligned} X^L&= LN_4(X^{t_2}) + MLP(LN_4(X^{t_2})) \end{aligned}$$

The $X^{t_1}$ and $X^{t_2}$ are temporary variables used to facilitate the description of these formulas.

The input of each encoder or decoder is some feature maps, but the Self Attention module in Swin Block3D needs to divide the feature maps into voxel patches, and then turn each voxel patch into a one-dimensional token in order to calculate the self-attention. After the self-attentive calculation, each token needs to be converted into the corresponding voxel patches, and finally, these voxel patches are stitched into the feature map. In general, the conversion between token and voxel patches is the dimensional transformation of the matrix. A voxel patch of dimension [*h*, *w*, *d*] is flattened to a one-dimensional token of length $h \times w \times d$, and a token of length $h \times w \times d$ can be transformed to a voxel patch of dimension [*h*, *w*, *d*] by matrix dimension transformation. We used the class named Rearrange in the einops [41] library to convert tokens and feature maps to each other.

#### Patch Merging3D and Patch Expanding3D

The function of the Patch Merging3D module mainly includes reducing the image spatial resolution and increasing the number of image channels. The structure of the Patch Expanding3D module, shown in Fig. 2c, is relatively simple, containing only a Conv3D layer and a LayerNorm [37] layer, while the functions of the Patch Expanding3D module are exactly the opposite of those of the Patch Merging3D module, mainly consisting of gradually restoring the image spatial resolution and reducing the number of image channels. As shown in Fig. 2d, this module contains a ConvTranspose3d layer and a LayerNorm layer.

The reason why these two modules use LayerNorm [37] instead of BatchNorm is as follows: 3D images generally occupy a relatively large amount of GPU memory, so during the experiment, the BatchSize parameter generally cannot be too large, otherwise, it will easily cause GPU memory overflow. However, in the case of BatchSize is relatively small using BatchNorm is not very meaningful.

#### Encoder and decoder

The internal details of the Encoder and Decoder are shown in Fig. 1. Each Encoder consists of a Patch Merging3D sub-module, a Conv Blocks3D sub-module, and more than one Swin Blocks 3D sub-module. The Patch Merging3D sub-module outputs a temporary feature image ($X^t$) after downsampling the input image or the feature image output from the previous Encoder. Both the Conv Blocks3D sub-module and the Swin Blocks3D sub-module get $X^t$ as input, where the Conv Blocks3D sub-module is used to learn short-distance dependency information in $X^t$, while the Swin Blocks3D sub-module is used to learn long-distance dependency information in $X^t$. A matrix addition operation on the output of the Conv Blocks3D and Swin Blocks3D sub-modules yields the feature image of this Encoder output.

The internal composition of the Decoder is similar to that of the Encoder, with the difference that there are two inputs given to the Decoder. The first input is the feature image output by the Encoder of the same level, and the second input is the feature image output by the Decoder of the previous level or the feature image output by the $Encoder_5$. The first input is added mainly to introduce the residual connection to avoid the gradient vanishing in the back-propagation process [42].

### Evaluation metrics

For each segmentation task, we used the dice coefficient [14] as an evaluation criterion, which is defined as follows:

12 $$\begin{aligned} Dice = \frac{2\Vert X \bigcap Y\Vert }{\Vert X\Vert + \Vert Y\Vert } = \frac{2TP}{2TP+FP+FN} \end{aligned}$$

where X is the prediction result of the models, Y is the ground truth, TP refers to the correctly classified tumor voxels, FN refers to the correctly classified non-tumor voxels, and FP refers to those voxels that are determined to be tumors by the model but are non-tumors in the ground truth. The dice coefficient is used to measure the similarity between the model prediction and the ground truth (GT), and its value ranges from 0 to 1. The closer the dice coefficient is to 1, the closer the prediction is to the GT.

### Experiments

#### Experimental conditions configuration

Swin Unet3D was implemented on Python 3.7.9, PyTorch 1.9.0 [43], and einops 0.3.2 [41]. We used an RTX3090 graphics card and an NVIDIA A100 graphics card to complete these experiments. To accelerate the training process of the model, we used the hybrid precision provided in PytorchLightning [44] for model training, inferences, and gradient accumulation techniques, thus disguising the expansion of the BatchSize. In all experiments, we used the Monai [45] framework to complete the preprocessing of the input images.

**Fig. 4:**
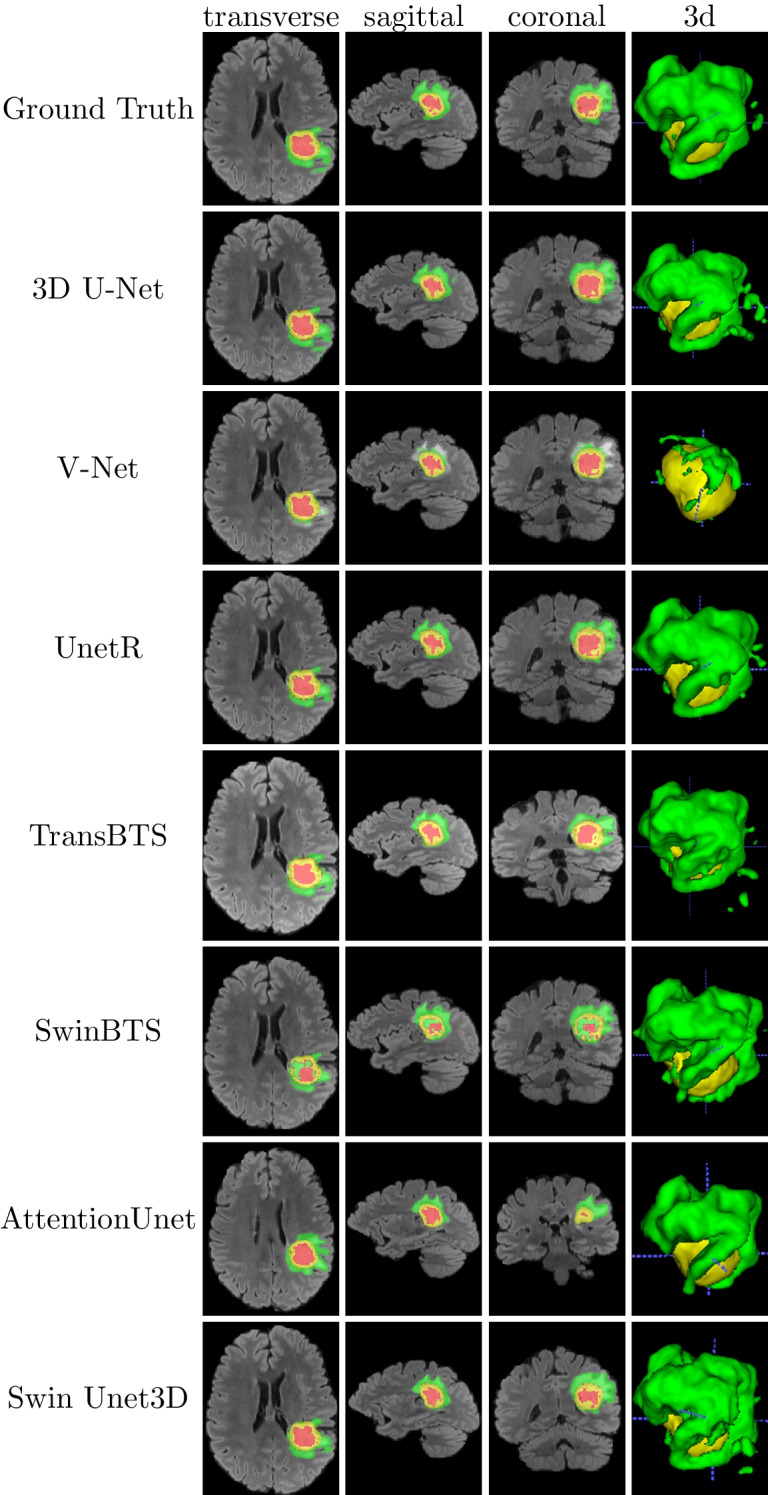
The segmentation results of each model were visualized on the validation dataset of Brats2021 using the ITK-SNAP [46] software. The red area in the figure shows the necrotic tumor core (NCR—label 1), the green area shows the peritumoral edematous/invaded tissue (ED—label 2), and the yellow area shows the GD-enhancing tumor (ET—label 4)

#### Experiment on the dataset of Brats2021 Challenge

The Brats2021 Challenge [33–35] dataset contains a total of 2000 MRI scans of glioma patients, with a training dataset size of 1251. Each patient’s MRI scan contains four contrasts: native T1-weighted, post-contrast T1-weighted (T1-GD), T2-weighted, and T2 Fluid-Attenuated Inversion Recovery (T2-Flair) images. All MRI scans have an image size of $240 \times 240 \times 155$ after interpolation to a resolution of $1\,mm^3$. Segmentation annotations for all of the patients included the GD-enhanced tumor (ET-Label 4), peritumoral edema/infiltrating tissue (ED-Label 2), and necrotic tumor core (NCR-Label 1).

To obtain better segmentation results and faster convergence of the model, we converted the multi-class labels into a multi-label segmentation task in the one-hot format, as follows: Label 2 was used to construct the enhancing tumor(ET), label 2 and label 4 were combined to construct the Tumor core(TC) channel, and label 1, label 2, and label 4 were merged to construct the Whole tumor (WT). The merge operation was implemented by a logical OR operation.

All models were trained and validated using the same hyperparameters, except for some individual model-specific hyperparameters, as detailed below. The data set was divided into training and validation sets in the ratio of 0.8:0.2, using a random seed of 42. The validation set was divided at the beginning of the training phase and was not involved in the training process. To make all experimental results reproducible, we also used random seeds with values equal to 42 for initializing the environments of PyTorch [43], PyTorch-lightning [44], Monai [45], and Cuda.

All models use the AdamW [47] optimizer and the DiceLoss [14] loss function, with the Batchsize being set to 1 and then disguised to expand the BatchSize to 16 using the gradient accumulation technique in PytorchLightning [44]. In the training stage, a region of size $128 \times 128 \times 128$ is randomly cropped from the original image and fed into the model for training. In the validation stage, we use the sliding window inference technique provided by Monai [45], with a sliding window size of [128, 128, 128] and an overlap region overlap parameter of 0.125. All models use the same learning rate of 3e-4. We also use the early stop technique in Pytorch-Lightning [44] to avoid overfitting the model on the training dataset. Figure 4 provides a comparison of the segmentation results among Ground Truth and the individual models.

**Fig. 5:**
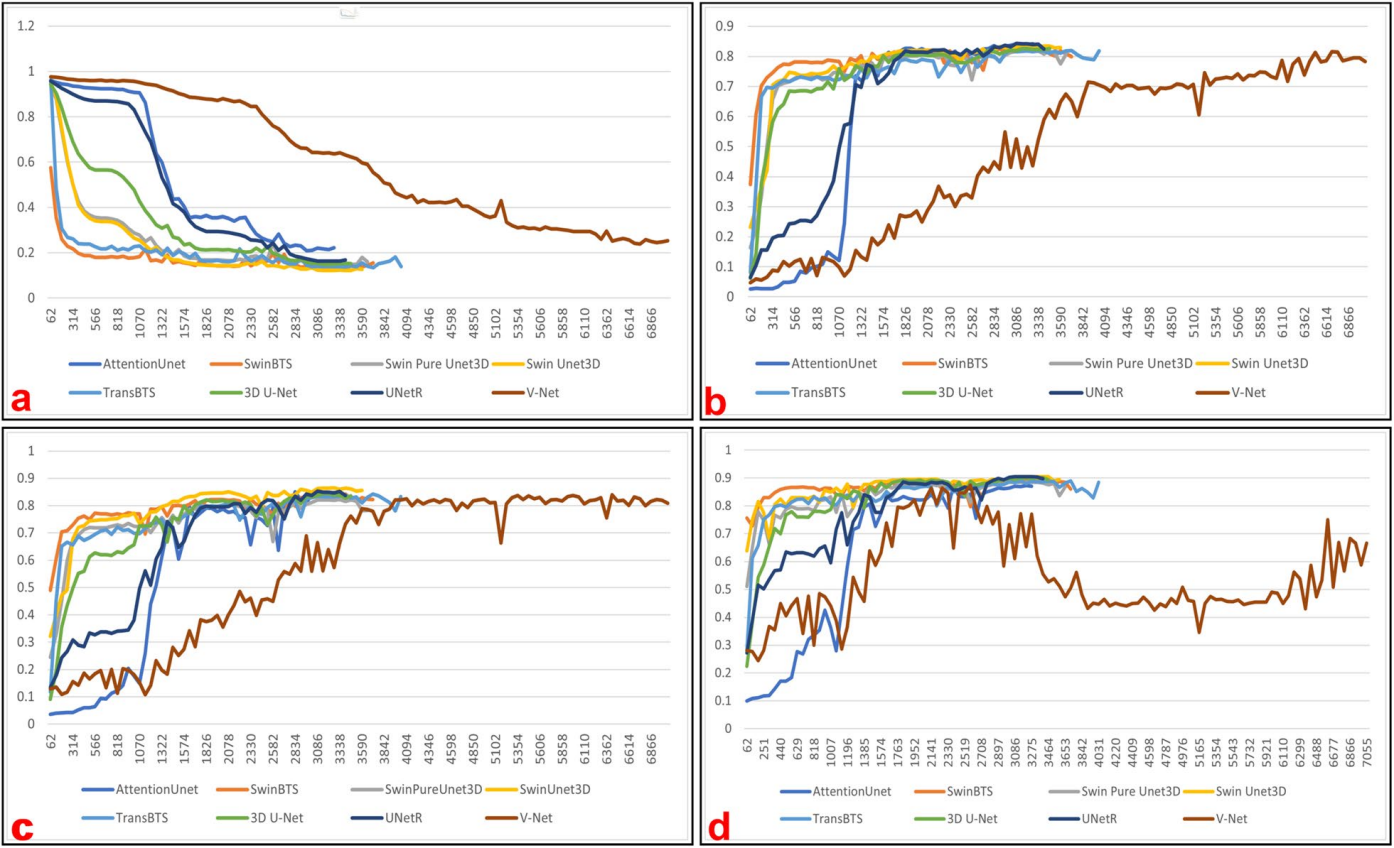
Variation curves of metrics for measuring the model fitting ability on the validation dataset of Brats2021: **a** mean loss; **b** dice coefficient on ET channel; **c** dice coefficient on TC channel; **d** dice coefficient on WT channel

#### Experiments on the dataset of Brats2018 Challenge

We also complete comparative experiments on Swin Unet3D and other models on the validation dataset of Brats2018 [33–35]. Brats2018 has the same segmentation regions and targets as Brats2021, but the dataset size is different. The training dataset for Brats2018 contains 285 MRI scans of patients with glioma.

**Fig. 6:**
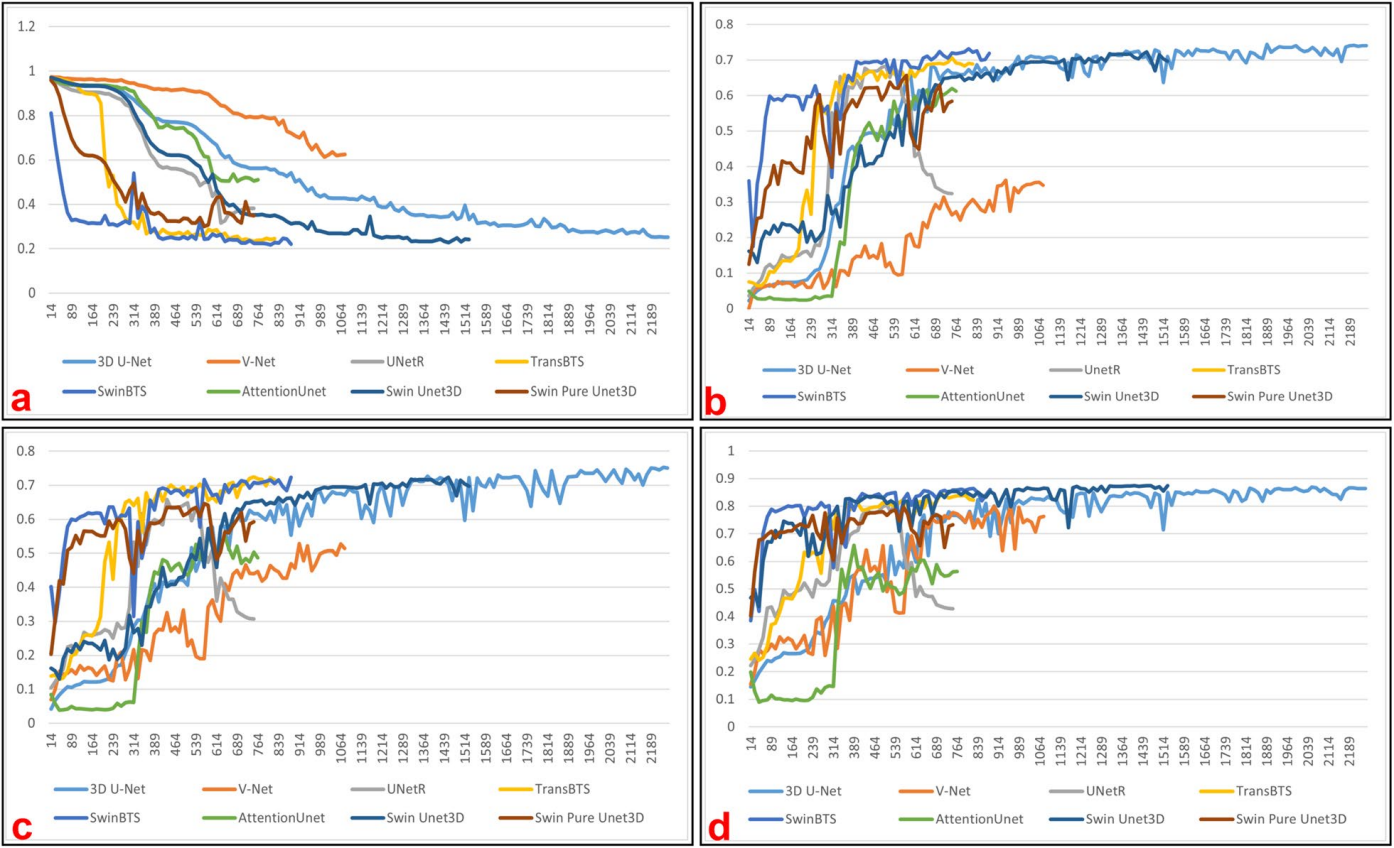
Variation curves of metrics for measuring the model fitting ability on the validation dataset of Brats2018: **a** mean loss; **b** dice coefficient on ET channel; **c** dice coefficient on TC channel; **d** dice coefficient on WT channel

And the experimental results are shown in Table 2, The change curves of relevant metrics can be obtained from Fig. 6. In the training stage of this experiment, we crop random image blocks of size $128 \times 128 \times 128$ from the input images and feed them into the network for training. In the inference stage, we use the sliding window body mechanism provided by the monai [45] framework, with the roi_size parameter set to [128, 128, 128] and the overlap parameter set to 0.125. In addition, the other parameters used in this experiment were completely inherited from Brats2021, which was done to test the fitting ability of Swin Unet3D more fully.

**Table 1:** Performance comparison of multiple models on the Brats2021 validation dataset and and the analysis of the significant differences between the performance of Swin Unet3D on the validation set and the performance of other models using the Wilcoxon sign test

Model name	Params	Params size	Mean dice			Significant difference		
	(M)	(MB)	ET	TC	WT	ET	TC	WT
3D U-Net	7.9	15.834	0.825	0.844	0.900	Yes	No	Yes
V-Net	45.6	182.432	0.815	0.840	0.751	No	Yes	Yes
UnetR	102	204.899	**0**.**842**	0.853	**0**.**905**	Yes	No	Yes
TransBTS	33.0	65.975	0.824	0.843	0.889	Yes	Yes	Yes
SwinBTS	35.7	71.394	0.828	0.843	0.896	Yes	Yes	Yes
AttentionUnet	23.6	47.257	0.841	0.851	0.870	No	No	No
Swin Pure Unet3D	33.6	67.163	0.817	0.822	0.885	Yes	Yes	Yes
Swin Unet3D	33.7	67.403	0.834	**0**.**866**	**0**.**905**	–	–	–

Bold values indicate the best metrics

**Table 2:** Performance comparison of multiple models on the Brats2018 validation dataset and the analysis of the significant differences between the performance of Swin Unet3D on the validation set and the performance of other models using the Wilcoxon sign test

Model name	Params	Params size	Mean dice			Significant difference		
	(M)	(MB)	ET	TC	WT	ET	TC	WT
3D U-Net	7.9	15.834	0.704	0.763	0.869	No	Yes	Yes
V-Net	45.6	91.216	0.361	0.528	0.801	Yes	Yes	Yes
UnetR	102	204.899	**0**.**743**	**0**.**767**	0.869	Yes	Yes	Yes
TransBTS	33.0	65.975	0.707	0.723	0.844	Yes	Yes	No
SwinBTS	15.7	34.411	0.732	0.717	0.863	No	No	No
AttentionUnet	23.6	47.257	0.613	0.550	0.658	Yes	Yes	Yes
Swin Pure Unet3D	33.6	67.163	0.657	0.646	0.797	Yes	Yes	Yes
Swin Unet3D	33.7	67.403	0.716	0.761	**0**.**874**	–	–	–

Bold values indicate the best metrics

## Result and discussion

### Result analysis

To explore the fitting ability of our model, we completed model training and segmentation performance validation on the datasets of Brats2018 and Brats2021. And we also conducted comparison experiments using 3D U-Net [9], V-Net [14], UnetR [31], TransBTS [30], SwinBTS [32], and AttentionUnet [16], with almost the same hyper-parameters for all experimental trials. As the number of training epochs increases, these models achieve the average dice coefficients on the validation dataset as shown in Figs. 5 and 6. From Table 1, it can be seen that the average dice coefficients achieved by our models on the validation dataset of Brats2021 are 0.834 (ET channel), 0.866 (TC channel), and 0.905 (WT channel), respectively. From Table 2, it can be seen that our model achieved average dice of 0.716 (ET channel), 0.761 (TC channel), and 0.874 (WT channel) on the Brats2018 dataset, respectively. Combining the results in Tables 1 and 2, we can tentatively conclude that our model achieves a better balance of model size and segmentation accuracy compared to other models. To explore the fitting ability of our model, we completed model training and segmentation performance validation on the datasets of Brats2018 and Brats2021. And we also conducted comparison experiments using 3D U-Net [9], V-Net [14], UnetR [31], TransBTS [30], SwinBTS [32], and AttentionUnet [16], with almost the same hyper-parameters for all experimental trials. As the number of training epochs increases, these models achieve the average dice coefficients on the validation dataset as shown in Figs. 5 and 6. From Table 1, it can be seen that the average dice coefficients achieved by our models on the validation dataset of Brats2021 are 0.840 (ET channel), 0.874 (TC channel), and 0.911 (WT channel), respectively. From Table 2, it can be seen that our model achieved average dice coefficients of 0.716 (ET channel), 0.761 (TC channel), and 0.874 (WT channel) on the validation dataset of Brats2018, respectively. Combining the results in Tables 1 and 2, we can tentatively conclude that our model achieves a better balance of model size and segmentation accuracy compared to other models.

### Discussion

**Fig. 7:**
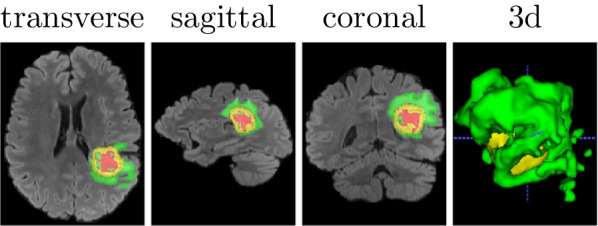
Visualization of segmentation results of Swin Pure Unet3D (Swin Unet3D implemented purely based on Swin Transformer)

We also tried to remove the Conv Blocks3D submodule from Swin Unet3D, and only the Swin Block3D submodule is used to complete the image feature extraction work, getting a model called Swin Pure Unet3D. As can be seen from Table 1 and Fig. 5, the average dice coefficients achieved by Swin Pure Unet3D on the validation dataset of Brats021 are consistently lower than those of Swin Unet3D. Figure 7 was used to visualize the prediction results of Swin Pure Unet3D, which uses MRI scans of the same patient as in Fig. 5 from the Brats2021 validation dataset. Although the difference between the average dice coefficient achieved by Swin Pure Unet3D and Swin Unet3D on the Brats2021 validation dataset is no more than 4.4%. This difference appears to be relatively small, however, it can be seen from Fig. 7 that many noise points appear in the prediction results of Swin Pure Unet3D, in contrast, there is no such phenomenon in Swin Unet3D, and the prediction results of Swin Unet3D are closer to Ground Truth. It can be seen from Table 2 and Fig. 6 that on the validation dataset of Brats2018, the difference between Swin Pure Unet3D and Swin Unet3D is much larger, with the largest dice coefficient difference reaching 7.7% (WT channel).

We also performed significance testing experiments, and Table 3 depicts the significance testing results on the validation dataset of Brats2021, while Table 4 shows the significance testing results on the validation dataset of Brats2018. We first saved the dice coefficients obtained for each model on the validation dataset to a CSV file, and then used the SciPy [48] library to perform significance analysis on the dice coefficients obtained for each model and the dice coefficients obtained for Swin Unet3D. The analysis results were kept in four valid digits. Together with the results shown in Tables 3 and 4, it can be seen that there is a sizable significant difference between Swin Pure Unet3D and Swin Unet3D on segmentation performance. This could, to some degree, indicate that the convolutional module can compensate for ViT’s inability to fit the image detail information well.

**Table 3:** On the validation dataset of Brats2021, we applied Wilcoxon Signed-Rank Test to the dice coefficients obtained from the comparison model and the dice scores obtained from Swin Unet3D, respectively

model name	P-value		
	ET	TC	WT
3D U-Net	0.0001	0.1073	0.0022
V-Net	0.0058	0	0.0115
UnetR	0.001	0.057	0
TransBTS	0	0	0
SwinBTS	0	0	0.0108
AttentionUnet	0.0079	0	0.0005
Swin Pure Unet3D	0	0	0

**Table 4:** On the validation dataset of Brats2018, we applied Wilcoxon Signed-Rank Test to the dice coefficients obtained from the comparison model and the dice scores obtained from Swin Unet3D, respectively

model name	P-value		
	ET	TC	WT
3D U-Net	0.2852	0.0133	0.0003
V-Net	0	0	0
UnetR	0	0	0
TransBTS	0.0038	0.0001	0.0559
SwinBTS	0.3714	0.3304	0.6393
AttentionUnet	0	0	0
Swin Pure Unet3D	0	0	0

## Conclusion

In this paper, we proposed a new 3D medical image segmentation model by adding the Swin Block3D module based on the Swin Transformer and Conv Block3D module based on CNN to each decoder and encoder of the model.

The Swin Block3D sub-module based on ViT is responsible for learning the global dependency information in the image, and the Conv Blocks3D sub-module based on convolution is responsible for learning the local dependency information of the image. Merging the dependency information learned by these two can ensure that all layers in Swin Unet3D will model the dependency information of the image well. Meanwhile, using jump connections in Swin Unet3D can mitigate the excessive loss of image information due to downsampling in the encoder. We have demonstrated the powerful fitting ability of the Swin Unet3D model through experiments on the Brats2021 Challenge and Brats2018 Challenge datasets. The results of the ablation experiments show that the Conv Blocks3D module and the Swin Block3D module can compensate for each other’s inherent deficiencies.

## Data Availability

The Brats2021 dataset used in this study can be found in the RSNA-ASNR-MICCAI Brain Tumor Segmentation (BraTS) Challenge 2021, http://braintumorsegmentation.org/. The dataset of Brats2018 used in this study can be found in the Paddle website,https://aistudio.baidu.com/aistudio/datasetdetail/64660.
